# Prohibit WP2 – systematic review of national guidelines in European countries

**DOI:** 10.1186/1753-6561-5-S6-P318

**Published:** 2011-06-29

**Authors:** M Martin, C Wilson, W Zingg, S Hansen, P Gastmeier, D Pittet, M Dettenkofer, PROHIBIT consortium

**Affiliations:** 1 University Medical Center Freiburg, Freiburg, Germany; 2 Hôpitaux Universitaires de Genève, Genève, Switzerland; 3 Charité - Universitätsmedizin Berlin, Berlin, Germany

## Introduction / objectives

The European Commission (FP-7) funded project PROHIBIT (Prevention of Hospital Infections by Intervention and Training) was established in 2010. WP2 of PROHIBIT aims to analyse existing national guidelines, practices in surveillance and public reporting of healthcare associated infections (HAI) in Europe.

## Methods

The ECDC-HAI surveillance National Contact Points (NCP) and experts in 34 countries (27 EU member states, whereby UK counts as 4 countries, Croatia, Iceland, Norway and Switzerland) were invited to complete a questionnaire and give information about existing national guidelines for prevention of surgical site infection (SSI), ventilator-associated pneumonia (VAP), catheter-associated urinary tract infection (CAUTI), catheter- associated blood stream infection (CA-BSI) and *C. difficile* infection (CDI). The NCPs were requested to state where the guidelines could be found in print or online. Stated websites were searched and compared to answers. In cases of mismatch NCPs were personally contacted for clarification.

## Results

End of March 2011 32 NCPs (94%) completed the questionnaire. 14 countries have guidelines on all 5 topics. 7 countries have 4 guidelines, 1 has 3 and 4 have 1. 5 countries have no national guidelines. Scientific level of supporting evidence and strength of recommendation are stated in 51% of the guidelines. In most countries identification of relevant documents was only possible by personal communication with NCPs. Scope, structure and detailing of recommendations vary widely.

## Conclusion

Evidence-based, national guidelines for prevention of HAI are still to be developed in many European countries. In the majority of countries finding the currently valid guidelines may be difficult for users.

## Disclosure of interest

None declared.

